# Transcriptome Analysis of Jojoba (*Simmondsia chinensis*) during Seed Development and Liquid Wax Ester Biosynthesis

**DOI:** 10.3390/plants9050588

**Published:** 2020-05-04

**Authors:** Saqer S. Alotaibi, Mona M. Elseehy, Bandar S. Aljuaid, Ahmed M. El-Shehawi

**Affiliations:** 1 Department of Biotechnology, Faculty of Science, Taif University, Taif 21974, Saudi Arabia; bssaljr@gmail.com; 2 Department of Genetics, Faculty of Agriculture, University of Alexandria, Alexandria 21545, Egypt; monaahmedma@yahoo.com

**Keywords:** jojoba, *Simmondsia chinensis*, transcriptome, wax ester, lipid metabolism, triglycerides, phospholipids, long chain fatty alcohol, very long chain fatty acid biosynthesis

## Abstract

Jojoba is one of the main two known plant source of natural liquid wax ester for use in various applications, including cosmetics, pharmaceuticals, and biofuel. Due to the lack of transcriptomic and genomic data on lipid biosynthesis and accumulation, molecular marker breeding has been used to improve jojoba oil production and quality. In the current study, the transcriptome of developing jojoba seeds was investigated using the Illunina NovaSeq 6000 system, 100 × 10^6^ paired end reads, an average length of 100 bp, and a sequence depth of 12 Gb per sample. A total of 176,106 unigenes were detected with an average contig length of 201 bp. Gene Ontology (GO) showed that the detected unigenes were distributed in the three GO groups biological processes (BP, 5.53%), cellular component (CC, 6.06%), and molecular functions (MF, 5.88%) and distributed in 67 functional groups. The lipid biosynthesis pathway was established based on the expression of lipid biosynthesis genes, fatty acid (FA) biosynthesis, FA desaturation, FA elongation, fatty alcohol biosynthesis, triacylglycerol (TAG) biosynthesis, phospholipid metabolism, wax ester biosynthesis, and lipid transfer and storage genes. The detection of these categories of genes confirms the presence of an efficient lipid biosynthesis and accumulation system in developing jojoba seeds. The results of this study will significantly enhance the current understanding of wax ester biology in jojoba seeds and open new routes for the improvement of jojoba oil production and quality through biotechnology applications.

## 1. Background

Global environmental conditions have been negatively impacted by the increasing consumption rate of fossil fuel. This issue has become an industrial concern at both local and international levels. One way to decrease the consumption of fossil fuels is to develop and use alternative biofuel sources, such as bioethanol and biodiesel. Jojoba (*Simmondsia chinensis*) is a tree that originated in Mexico (Sonora Desert) and USA (California) and can be used for the production of economic biofuel. Jojoba is currently being cultivated in many parts of the world, including Mexico, Australia, India, Saudi Arabia, Tunisia, and Egypt. Jojoba produces about 65% oil in its seeds with specific characteristics known as liquid wax. The oil from a jojoba seed is different from other plant oil (triglycerides) in that it is mainly composed of liquid wax esters (long chain fatty acid esterified to a long chain fatty alcohol) [1,2,3]. The United States is the main producer of jojoba oil [4]. Previously, the main source of wax ester was the heads of whales’ sperms, but whale hunting has been banned since 1986 [5]. Currently, the main plant sources for liquid wax ester are jojoba seed oil and the palm wax obtained from *Copernicia prunifera* [6].

Several studies have noted the unique characteristics of jojoba oil that makes it particularly useful in cosmetics, lubrication, and the biofuel industry [7,8,9]. The texture and durability of jojoba oil makes it preferable for skincare, pharmaceuticals, as a substitute for synthetic polymers, and as a natural raw material for biofuel production [10]. It also has anti-inflammatory, antimicrobial [11], antifungal [12], and antioxidant [4] activities. The jojoba seed remaining after oil extraction is considered as a cheap high-energy feed component [13].

Wax esters are chemically produced using immobilised lipases that mainly depend on the chemical synthesis of fatty alcohols that dramatically raise the cost of production [14,15]. Most biodiesel is currently produced using vegetable oils by the esterification of oil to alcohol molecules with the help of a catalyst. This increases the demand for vegetable oils that would otherwise be consumed by humans. Several reports have suggested the use of jojoba oil for biofuel production to decrease the demand for other edible oils. Methyl esters were synthesized from jojoba oil producing a wax ester yield of 83.5% at an operational temperature of 25 °C [8]. Biodiesel was produced through the acetylation of the fatty alcohol of jojoba. The biodiesel produced had similar properties to jojobyl methyl esters (JME) and other biodiesel fuels [16]. Recently, biodiesel was produced from jojoba oil using calcium glyseroxide as a catalyst [17]. Biodiesel has also been developed from jojoba oil by transesterification, and its energy balance was investigated by estimating its net energy balance (NEB) and net energy ratio (NER). It had a NEB and a NER of 28.9 and 2.16 MJ/L, respectively. The greenhouse gas (GHG) emission of the produced biodiesel was 66 g CO2/MJ of biodiesel [18].

Jojoba wax ester has been produced in *E. coli* by metabolic engineering using *E. coli* strain expressing jojoba fatty alcohol reductase (FAR) and *Acinetobacter baylyi* wax ester synthase (WS). This study experimentally proved that engineered microorganisms could be used to produce and accumulate natural wax esters in oil bodies in their cytoplasm [15].

Transcriptome sequencing (*de novo* or resequencing) is a powerful approach to investigate changes in global gene expression during plant growth and development. It has been employed to study gene expression during seed germination in *Phelipanche aegyptiaca* [19], floral initiation in *Pongamia pinnata* [20], seed development in pecan trees (*Carya illinoinensis*) [21], seed development in *Camelina sativa* [22], seed development in *Euryale ferox* [23], and seed development in *Millettia pinnata* [24]. Transcriptome analysis has also been employed to study oil biosynthesis in *Pongamia pinnata* [20], oil content and fatty acid composition in *Camellia oleifera* [25], lipid metabolism in pecan trees seeds (*Carya illinoinensis*) seeds (21), alpha-linolenic acid synthesis in tree peony seeds [26], triacylglycerol biosynthesis in *Camelina sativa* [22], oil accumulation in *Millettia pinnata* [24], and the biosynthesis of unsaturated fatty acids in *Perilla frutescens* [27].

Jojoba has been extensively studied using molecular markers to investigate the genetic diversity among varieties to improve oil production and to detect the sex of the plant. In these studies, a wide range of molecular markers were employed, including ISSR [28,29], AFLP [30], SCAR [3], SCoT [31], and protein-based biomarkers [32]. The focus of these studies was to enhance jojoba oil production through biodiversity and breeding. One limitation of jojoba oil use in medical and cosmetic applications is the high cost [14]; therefore, lowering its production cost is a valuable objective for the medicinal, cosmetic, and biofuel industries.

Previous studies have focussed on the genetic diversity in jojoba using molecular markers to enhance oil production through breeding, but there has not yet been a comprehensive molecular study on this economic plant. No studies have done a transcriptome analysis of the developing jojoba seeds with a focus on the biosynthesis of long chain fatty acids and long chain fatty alcohol, which are essential processes for wax ester biosynthesis during seed development. Therefore, the aim of this study is to investigate the jojoba transcriptome during seed development. This will provide a foundation for molecular biology and biotechnology studies to improve oil production and identify the main genes responsible for oil component biosynthesis. The results of this study will enhance our understanding of the molecular biology of the jojoba seed and how this molecular data can be used to improve jojoba oil production through biotechnology applications.

## 2. Results

To investigate the global gene expression during jojoba seed development, seed samples were collected during seed development and used for de novo transcriptome analysis. As this is the first transcriptome study of jojoba, all samples were assembled to reconstruct sequences of the transcriptome without a reference genome. The assembled contigs were merged in a set of non-redundant unique transcripts and then clustered into unigenes with a 200 bp minimum length cut-off. The clustered unigenes were annotated by blasting them against various public databases using BLASTN and BLASTX. The databases included KEGG, NT, Pfam, GO, NR, UniProt, and EggNOG. Annotation included the prediction of ORFs to identify protein-coding sequences in the unigenes. Additionally, the unigenes were used for read alignment and the abundance was estimated as read count and FPKM.

### 2.1. De Novo Assembly of the Jojoba Transcriptome

For the initial assembled contigs, 176,106 genes and 233,291 transcripts were detected with an average contig length of 710 bp and 165,664,392 total assembled bases. For the longest contigs, 176,106 genes and 176,106 transcripts were detected with an average contig length of 589 bp and 103,727,640 bases assembled. For unigene contigs, 167,684 genes and 167,684 transcripts were detected with an average contig length of 600 bp and 100,770,670 total bases assembled. The GC content was approximately 40% in all types of contigs (Supplementary Materials Table S1).

After trimming, the total number of read bases ranged from 13 × 10^9^ to 14 × 10^9^ bases, the total reads ranged from 137.5 × 10^6^ to × 143.8 10^6^ bases, and the GC content was about 50%. The trimmed reads were clustered into transcript unigenes and merged into one file to construct the combined de novo transcriptome reference. In the combined merge, 176,106 unigenes and 176,106 transcripts were detected with 17,658 bp maximum contig length, 201 bp minimum contig length, 348.0 bp median contig length, and 589.01 bp average contig length (Supplementary Materials Table S2).

### 2.2. Abundance Estimation

Trimmed reads for each sample were aligned to the assembled reference using the program Bowtie. For the differentially expressed gene analysis, the abundances of unigenes across the samples were estimated into read count by the RSEM algorithm or FPKM. The number of processed reads ranged from 137.5 × 10^6^ to 143.8 × 10^6^, the mapped reads ranged from 116 × 10^6^ (84.9%) to 121 × 10^6^ (84.5%), while unmapped reads ranged from 20.8 × 10^6^ (15.2%) to 22.3 × 10^6^ (15.5%).

### 2.3. ORF Prediction

ORFs were predicted using TransDecoder with a minimum length of 100 amino acids to detect expected coding regions in the transcripts. Among the 167,684 detected unigenes in this study, only 20,162 (12%) had predicted ORFs. These ORFs were distributed among two groups: 18,082 (89.7%) had single ORFs and 2080 (10.3%) had multiple ORFs. The number of total predicted ORFs was 22,579, of which 13123 (58.12%) were complete ORFs, 2111 (9.35%) were internal ORFs, 5813 (25.75%) were 5′ partial ORFs, and 1532 (6.79%) were 3′ partial ORFs (Supplementary Materials Table S3).

### 2.4. Annotation

The clustered unigenes were annotated against various public databases. The annotation results showed that about 24.66% of the total unigenes were annotated to more than at least one database (41357 unigenes of 167684 total unigenes). The annotation percentages of the clustered unigenes were 23.68%, 9.58%, 21.74%, 16.2%, 23.46%, 14.59%, and 17.47% for KEGG, NT, EggNOG, Pfam, NR, UniProt, and GO databases, respectively (Supplementary Materials Table S4).

### 2.5. Annotation on GO Database

**Figure 1:**
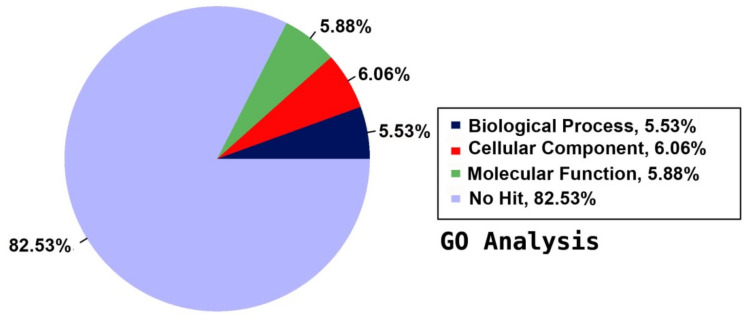
Summary of Gene Ontology (GO) annotation using the detected unigenes of the jojoba transcriptome of developing seeds.

**Figure 2:**
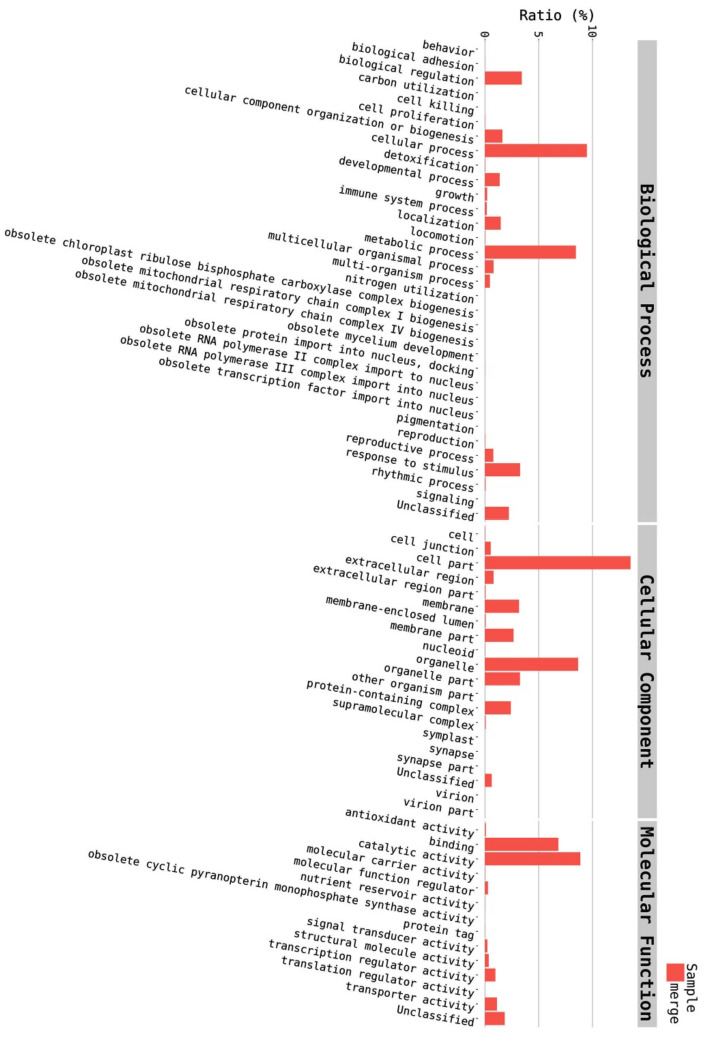
Distribution of annotated unigenes into the 67 functional groups of biological process (BP), cellular component (CC), and molecular function (MF).

For a functional prediction of the unigenes, they were annotated against the GO database to classify the annotated unigenes using BLASTX of DIAMOND with an E-value cut-off of 1.0 × 10^−5^. The detected unigenes were categorised in three main groups according to their contribution to the plant’s functions: biological process (BP), cellular component (CC), and molecular function (MF). The GO annotation indicated that about 7.47% of the total 167,684 unigenes had GO assignments in the assembled reference transcriptome. The annotated unigenes were distributed among the three main categories as 5.53% BP, 6.06% CC, and 5.88% MF, and the majority of the unigenes (82.53%) were not annotated to any functional group (Figure 1). The 5.53% BP, 6.06% CC, and 5.88% MF (Figure 1) were distributed among 33 BP terms, 20 CC terms, and 14 MF terms consecutively, giving a total of 67 functional groups (Figure 2).

**Figure 3:**
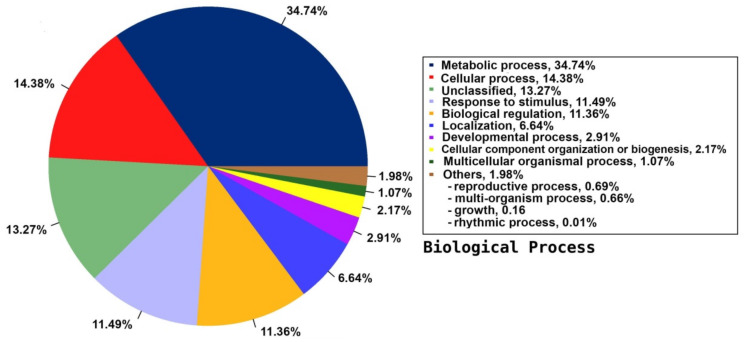
Distribution of the annotated BP to the GO database using the detected unigenes of the jojoba transcriptome of developing seeds.

**Figure 4:**
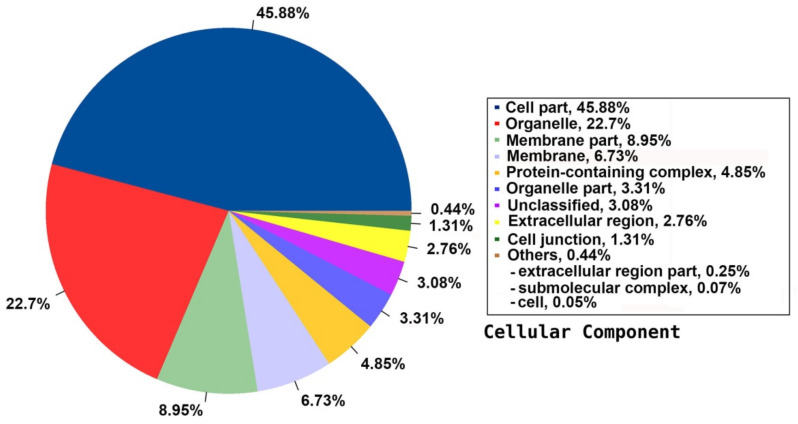
Distribution of the annotated CC to the GO database using the detected unigenes of the jojoba transcriptome of developing seeds.

**Figure 5:**
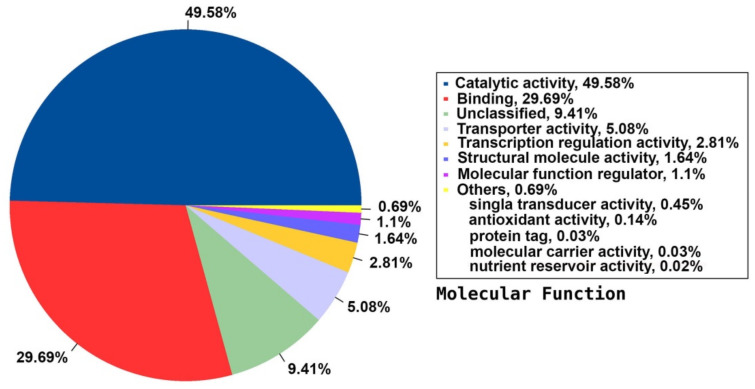
Distribution of the annotated MF to the GO database using the detected unigenes of the jojoba transcriptome of developing seeds.

Among the 33 BPs, metabolic processes was the major category (34.74%) followed by cellular processes (14.38%), response to stimulus (11.49%), and biological regulation (11.36%). Other BPs included localisation (6.64%), developmental process (2.91%), cellular component organisation or biogenesis (2.17%), and multicellular organismal process (1.07%) (Figure 3). The CC group of GO was distributed among 20 cell components, including cell part (45.88%), organelle (22.7%), membrane part (8.95%), membrane (6.73%), protein-containing complex (4.85%), organelle part (3.31%), and cell junction (1.31%) (Figure 4). Among the 14 MFs, catalytic activity (49.58%) was the most predominant category, followed by binding (29.69%). Other MF groups included transporter activity (5.88%), transcription regulator activity (2.81%), structural molecule activity (1.6%), molecular function regulator (1.1%), signal transducer activity (0.45%), and molecular carrier activity (0.03%) (Figure 5).

### 2.6. EggNOG Analysis

**Figure 6:**
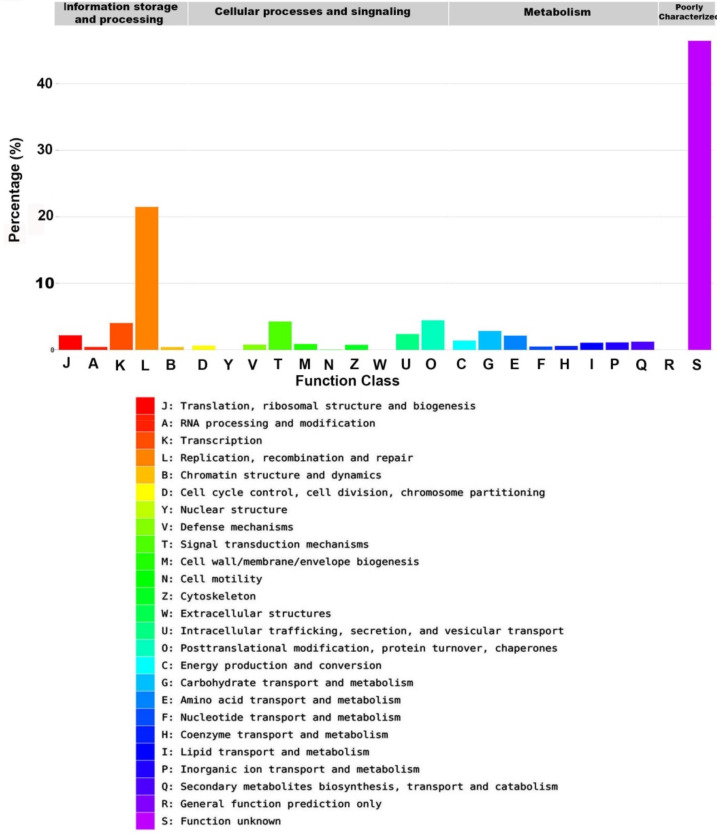
The annotated sequence percentage of detected unigenes against the Evolutionary genealogy of genes: Non-supervised Orthologous Groups (EggNOG) database.

To identify orthologous proteins for the annotated unigenes in eukaryotic orthologous groups (KOG), clusters of orthologous groups (COGs), and non-supervised orthologous groups (NOGs), BLASTX of DIAMOND was used with an E-value cut-off of 1.0 × 10^−5^ on the EggNOG database. The annotated unigenes were mapped to the corresponding orthologous groups in the EggNOG database. The EggNOG database focuses on the predicted function of unigenes based on their orthologs in other species. The annotated unigenes were separated into three main groups—information storage and processing, cellular processes and signalling, and metabolism—which are distributed in 25 functional groups in the EggNOG database (Figure 6).

The most predominant functional group in the EggNOG database was the replication, recombination, and repair group (7802 unigenes, 21.48%). Other major groups included transcription (1462, 4.03%), signal transduction mechanisms (1542, 4.25%), posttranslational modification, protein turnover, and chaperones (1612, 4.44%), carbohydrate transport and metabolism (1031, 2.84%), intracellular trafficking, secretion, and vesicular transport (866, 2.38%), translation, ribosomal structure and biogenesis (797, 2.19%), amino acid transport and metabolism (778, 2.14%), energy production and conversion (504, 1.39%), secondary metabolites biosynthesis, transport and catabolism (446, 1.23%), and inorganic ion transport and metabolism (407, 1.12%). Lipid transport and metabolism, which is the most related group to this study, represented 390 unigenes (1.07%). About 16,866 (46.44%) unigenes were not annotated in the EggNOG database (Figure 6, Table 1).

### 2.7. Gene Expression Analysis

#### 2.7.1. Gene Expression of Lipid Biosynthesis Genes

**Figure 7:**
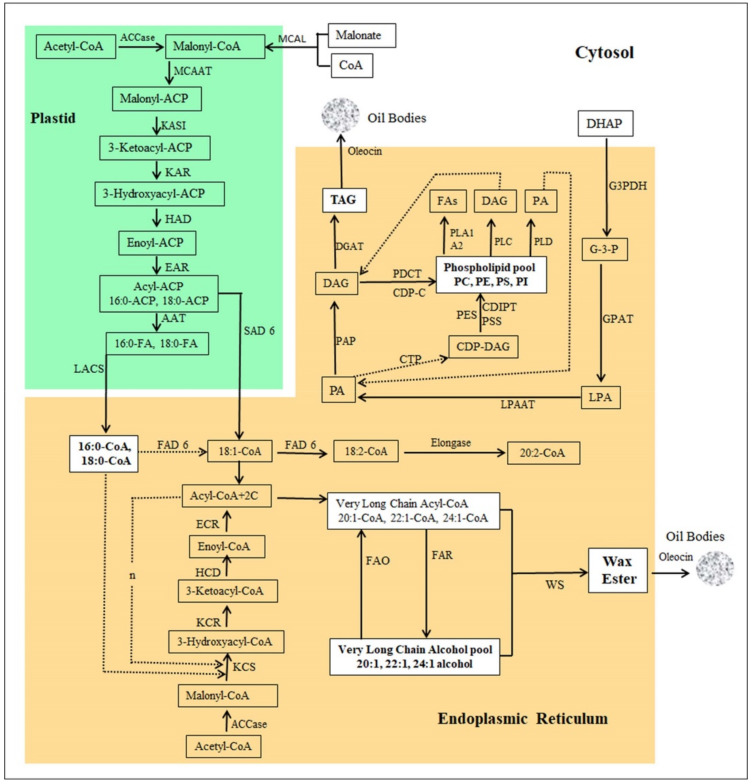
Pathway of Jojoba wax ester biosynthesis. MCAL: malonate-CoA ligase, ACC: acetyl-CoA carboxylase, MCAAT: malonyl-CoA acyl carrier protein (ACP) transacylase, ACAT: acetyl-CoA:ACP acetyltransferase, KASI: 3-ketoacyl-ACP synthase I, KAR: 3-ketoacyl-ACP reductase, HAD: 3-hydroxyacyl-ACP dehydratase, EAR: enoyl-ACP reductase, AAT: acyl-ACP thioesterase, LACS: long chain acyl-CoA synthase, KCS: 3-ketoacyl-CoA synthase (Elongase), KCR: 3-ketoacyl-CoA reductase, HCD: 3-hydroxyacyl-CoA dehydratase, ECR: enoyl-CoA reductase, SAD6: stearoyl-[acyl-carrier-protein] 9-desaturase, FAD6: omega-6 fatty acid desaturase, FAR: fatty acyl-CoA reductase, FAO: fatty alcohol oxidase, WS: wax ester synthase, elongase, G3PDH: glycerol-3-phosphate dehydrogenase, GPAT: glycerol-3-phosphate acyltransferase, LPAAT: 1-acyl-sn-glycerol-3-phosphate acyltransferase, PAP: phosphatidate phosphatase, DGAT: diacylglycerol O-acyltransferase, CTP: phosphatidate cytidylyltransferase, CDIPT: CDP-diacylglycerol--inositol 3-phosphatidyltransferase, PSS: CDP-diacylglycerol-serine O-phosphatidyltransferase, PES: N-acylphosphatidylethanolamine synthase, PDCT: phosphatidylcholine: diacylglycerol cholinephosphotransferase, PLA1: phospholipase A1, PLA2: phospholipase A2, PLC: phospholipase C, PLD: phospholipase D, DHAP: dihydroxyacetone phosphate, G3P: glyceraldehyde 3-phosphate, LPA: lysophosphatidic acid, PA: phosphatidic acid, DAG: diacylglycerol, TAG: triglycerides, CDP-DAG: CDP-diacylglycerol. CDP-C: CDP-choline.

Gene expression level was estimated across the samples as the unigene read count and FPKM. EggNOG annotation showed that 1.07% (390) of the annotated unigenes was related to lipid transport and metabolism. In jojoba, lipid metabolism generally includes de novo fatty acid (FA) biosynthesis, FA elongation, FA desaturation, long chain fatty alcohol (LCFA) biosynthesis, triacylglycerol (TAG) metabolism, and wax ester biosynthesis. In this study, all key enzymes and proteins involved in these main lipid biosynthesis processes were represented (Table 2, Figure 7).

#### 2.7.2. Fatty Acid Biosynthesis

Eight different genes for the de novo biosynthesis of fatty acids were detected in our transcriptome, represented by various numbers of unigenes with an overall FPKM range of 0.35–264.77. The highest FPKM values of fatty acid synthesis were 138.28, 152.71, 264.77, 198.16, 254.27, 174.01, and 21.47 for malonate CoA ligase (MCAL), malonyl CoA-acyl carrier protein transacylase (MCAAT), 3-ketoacyl acyl carrier protein (ACP) synthase I (KASI), 3-oxoacyl-[acyl-carrier-protein] reductase (KAR), 3-hydroxyacyl-[acyl-carrier-protein] dehydratase (HAD), enoyl-[acyl-carrier-protein] reductase (EAR), and palmitoyl-acyl carrier protein thioesterase (AAT, FATB), respectively (Table 2) (FPKM range, 21.47 to 264.77). Acetyl-CoA carboxylase (ACCase) carries the rate-limiting step in fatty acid biosynthesis. The enzyme complex has four subunits: biotin carboxylase (BC), carboxyl transferase α (CT-α), carboxyl transferase β (CT-β), and biotin carboxyl carrier protein (BCCP). The enzyme’s four subunits were highly expressed during the jojoba seed development with FPKM values of 195.99, 88.75, 4.95, and 229.71 for BC, α-CT, β-CT, and BCCP, respectively (Table 2). Long-chain acyl-CoA synthetase (LACS) transfers the acyl group from acyl-ACP at the end of their biosynthesis in the chloroplast into CoA to produce acyl-CoA and then transports them to the ER for chain elongation. Eleven unigenes were detected for LACS in the jojoba transcriptome with an FPKM of 3.21 to 95.33, indicating a high level of expression (Table 2, Figure 7).

#### 2.7.3. Fatty Acid Desaturation

Several fatty acid desaturases were detected in this study. Some of them are located in the endoplasmic reticulum (ER), including delta (12)-acyl-lipid-desaturase (FAD2) (FPKM, 69.07), omega-6 fatty acid desaturase (FAD6) (FPKM, 19.88), delta (8)-fatty-acid desaturase (SLD) (FPKM, 33.31), delta-7 desaturase (STE) (FPKM, 50.69), and sphingolipid delta (4)-desaturase DES1 (SLD) (FPKM, 8.87). Other desaturases are located in the plastid, including fatty acid desaturase 4 (FAD4) (FPKM, 2.05), palmitoyl-monogalactosyldiacylglycerol delta-7 desaturase (FAD5) (FPKM, 33.65), omega-6 fatty acid desaturase (FAD6) (FPKM, 8.42), and stearoyl-[acyl-carrier-protein] 9-desaturase (SAD6) (FPKM, 1369.99). SAD6 had one of the highest FPKM in this study. It converts stearoyl-ACP to oleoyl-ACP by introducing a *cis* double bond between carbons 9 and 10 of the acyl chain (Table 2, Figure 7).

#### 2.7.4. Fatty acid Elongation

Very long-chain fatty acids (VLCFAs) are the main substrate for wax ester biosynthesis in jojoba seeds. VLCFAs are synthesised by the enzyme complex elongase, which carries out the cyclic addition of 2C through its four subunits in the ER membrane. The subunits of elongase complex were highly expressed in the developing jojoba seeds. Their FPKM values were 917.77, 402.24, 0.27, and 76.33 for 3-ketoacyl-CoA synthase (KCS), 3-ketoacyl-CoA reductase (KCR), 3-hydroxyacyl-CoA dehydratase (HCD), and enoyl-CoA reductase (ECR), respectively (Table 2, Figure 7).

#### 2.7.5. Fatty Alcohol Synthesis and Oxidation

LCFA biosynthesis is a critical step for wax ester biosynthesis. Ten unigenes for alcohol-forming fatty acyl-CoA reductase (FAR) with a FPKM range of 0.56 to 1613.05 were detected in this study. It is noteworthy that FAR had one of the highest FPKM values among the lipid metabolism enzymes in this study. It catalyses the conversion of a long-chain acyl-CoA into long-chain alcohol, which is the second component of wax ester precursors. This enzyme had previously been isolated from jojoba [33]. LCFAs are oxidized into fatty acyl-CoA by the long chain fatty alcohol oxidase (FAO). Three unigenes were detected for FAO with the highest FPKM value of 39.02. FAR and FAO keep the balance between VLCFAs and LCFA to maintain wax ester biosynthesis during seed development.

#### 2.7.6. Triacylglycerol (TGA) Metabolism

Five enzymes involved in the TGA biosynthesis were detected: glycerol-3-phosphate dehydrogenase (G3PDH), glycerol-3-phosphate acyltransferase (GPAT), 1-acylglycerol-3-phosphate O-acyltransferase (LPAAT), phosphatidate phosphatase (PAP), and diacylglycerol O-acyltransferase (DGAT). These enzymes had FPKM values of 60.72, 13.59, 125.06, 24.62, and 16.53, respectively (Table 2). Three enzymes for acylglycerol lipases were detected: monoacylglycerol lipase (MAGL), mon-/di-acylglycerol lipase (DAGL), and triacylglycerol lipase (SDP, TAGL), with FPKM values of 15.34, 10.15, and 32.09, respectively (Table 2, Figure 7).

#### 2.7.7. Phospholipid Metabolism

Five enzymes for phospholipid synthesis were detected: CDP-diacylglycerol-inositol 3-phosphatidyltransferase 1 (CDIPT), CDP-diacylglycerol-serine O-phosphatidyltransferase 1 (PSS), N-acylphosphatidylethanolamine synthase (PES), phosphatidylcholine:diacylglycerol cholinephosphotransferase 1 (PDCT), and phosphatidate cytidylyltransferase 1 (CTP, CDS) with FPKM values of 24.10, 7.99, 6.6, 11.93, and 20.11, respectively (Table 2). Phospholipases hydrolyse phospholipids and release fatty acids and other lipophilic compounds. Six types of phospholipases were detected in this study, including phospholipase A1 (PLA1), phospholipase A2 (PLA2), phospholipase C (PLC), phospholipase D (PLD), phospholipase SGR2 (PLSGR2), and lysophospholipase BODYGUARD 3 (BDG3) with FPKMs of 3.8, 159.08, 35.9, 127.73, 13.24, and 42.11, respectively (Table 2, Figure 7).

#### 2.7.8. Wax ester Biosynthesis

WS is the final and most important enzyme for wax biosynthesis. It links the LCFAs with the VLCFAs to form the wax ester. Two different forms of WS were detected in the ER: WS and O-acyltransferase (WSD1), with FPKM values of 101.99 and 32.29, respectively (Table 2, Figure 7).

#### 2.7.9. Lipid Transfer and Storage Proteins/Enzymes

Five lipid transfer and storage proteins were detected: acyl carrier protein (ACP), acyl-CoA-binding protein (ACBP), non-specific lipid-transfer protein (nsLTPs), lipid-transfer protein DIR1 (LTP), and oleosin (OL, 8.2 kDa) with FPKMs of 1036.72, 244.44, 9748.18, 5214.36, and 3779.71, respectively (Table 2). This group includes the highest expressed genes in the jojoba transcriptome. For example, nsLTPs are the highest with an FPKM of 9748.18, LTP is next with 5214.36, and OL follows with an FPKM value of 3779.71 (Table 2).

## 3. Discussion

Jojoba is the main plant source for natural liquid wax ester. The available genomic information about jojoba is very scarce in public databases, especially the transcriptome of developing seeds, which is the most important stage of wax ester biosynthesis. Data obtained from transcriptome analysis of jojoba developing seeds were employed to establish the lipid biosynthetic pathway during seed development with an emphasis on the VLCFAs and LCFAs (the main components of wax ester) in jojoba seeds. The focus of this study was the detection of lipid biosynthesis genes and the interconnection between the functions of this gene network in the coordination of lipid biosynthesis.

It has been reported previously that the main source for liquid wax ester was from the heads of sperm whales, where it was believed to regulate their buoyancy [15], and from the oil of jojoba seeds [5,34,35]. Since whale hunting was banned, the only two known sources for liquid wax ester are jojoba oil and palm wax, or *carnauba* from *Copernicia prunifera* [6]. This makes this study the first de novo transcriptome analysis of liquid wax ester-producing seeds.

The assembled transcriptome detected 167684 unigenes were much higher than the detected unigenes in the de novo transcriptome analysis of developing seeds in *Camellia oleifera* (77052), [25], *Euryale ferox* (85006), [23], and *Carya illinoinensis* (82155), [21]. In this study, the GC content was about 40%, which was lower than the GC content in seeds of *Camellia oleifera* (47–49%) [25], *Pongamia pinnata* (48%) [20], *Euryale ferox* (48.8%) [23], and *Carya illinoinensis* (42.75%) [21]. Our data showed that 24.66% (41375) of the detected unigenes were annotated to at least one database. This was lower than the annotation percentage of unigenes in the seeds of *Camellia oleifera* (67.12%, 51725) [25], *Pongamia pinnata* (45%, 16146) [20], and pecan (39.56%) [21]. The lower annotation percentage in our study was due to the higher number of detected unigenes compared to the other studies.

### 3.1. Lipid Biosynthesis Gene Expression Profiling

The genes involved in lipid transport and metabolism were the focus of this study because they relate directly or indirectly to wax ester biosynthesis in jojoba seeds. Gene expression was estimated as the read count of unigenes or FPKM. About 390 (1.07%) unigenes were related to lipid transport and metabolism in this study. This was lower than the detected unigenes for lipid transport and metabolism (889, 3.8%) in the transcriptome of the developing seeds of pecan trees [21]. These discrepancies in transcriptome sequencing statistics indicate the high complexity, the diversity, and the plant-dependence of sequencing data. The lipid transport and metabolism genes were distributed among de novo FA biosynthesis, FA desaturation, FA elongation, fatty alcohol biosynthesis, TGA metabolism, phospholipid metabolism, wax ester biosynthesis, and lipid transfer and storage proteins/enzymes.

De novo synthesis of fatty acids (C:16–C:18) occurs in the plastids, which include palmitic, stearic and oleic acids [36]. Very long-chain fatty acids (VLCFAs) (C > 20) are synthesised in the endoplasmic reticulum (ER) by chain elongation of C:16–CoA and C:18–CoA that were initially synthesised in the plastids [37]. Phospholipids, including phosphatidylcholine (PC), phosphatidylethanolamine (PE), phosphatidylserine (PS), and phosphatidylinositol (PI), are synthesised in the ER. In plants, TAGs are synthesised in the ER [38] and are stored in oil bodies as an energy source for seed germination [39].

### 3.2. Fatty Acid Biosynthesis

Unigenes for the main eight enzymes involved in FA biosynthesis were detected in our study. MCAL was detected with an FPKM of 138.28. It is interesting that this enzyme was not detected in similar studies of transcriptome analysis during oil seed development. It provides an additional route for the production of malonyl-CoA, which is the main precursor for FA biosynthesis. The other seven genes for FA biosynthesis were detected in this study with different expression levels and number of unigenes, but the highest expression level among them ranged from an FPKM of 21.47 to 264.77.

The rate-limiting enzyme of FA biosynthesis is ACCase. The multisubunit form of ACCase was detected in our transcriptome. Three subunits, BC, CT-α, BCCP, were highly expressed during seed development with FPKM values of 195.99, 88.75, and 229.71, respectively, whereas the fourth subunit CT-β showed lower expression with an FPKM value of 4.95 (Table 2). A low expression level of CT-β was previously reported during the seed development of *Perilla frutescens* [27]. The expression of the same four subunits was reported in similar studies on *Arbidopsis* [40] and *B. napus* [41]. Fourteen unigenes were detected for ACCase and its subunits in the transcriptome of *Millettia pinnata* developing seeds [24]. Two types of ACCase were reported in *C. merolae* algae; the multifunctional type is encoded by nuclear genes and is located in the cytosol, whereas the multisubunit type is encoded by nuclear and plastid genes and is located in the plastid [36]. The upregulation of ACCase definitely enhances the composition and content of FAs in seeds [27,42], thus a high level of ACCase expression during jojoba seed development would enhance the accumulation of more substrate for de novo FA biosynthesis.

The enzymes MCAAT, KASI, KAR, HAD, and EAR use malonyl-CoA through a series of condensation reactions to form acyl-ACP (C:16, C:18). This group of enzymes was highly expressed with FPKM values of 152.71, 264.77, 198.16, 254.27, and 174.01, respectively (Table 2). The high expression level of this group is consistent with previously reported data [27,43]. In the *Millettia pinnata* transcriptome, MCAAT, KAR, and EAR had two unigenes, whereas KASI and HAD had one detected unigene [24]. Fatty acid biosynthesis genes were also detected in the *Camelina sativa* seed transcriptome [22].

FATA and FATB are responsible for releasing free FAs from the ACP group after their biosynthesis. FATA works on the unsaturated FAs, while FATB works on the saturated FAs to produce palmitic acid (16:0) and stearic acid (18:0). In this study, FATB (AAT) was highly expressed with an FPKM value of 21.47 (Table 2), whereas FATA was not detected. FATB was detected in the transcriptome of developing seeds of *B. napus* (four unigenes) [43], *Millettia pinnata* (two unigenes) [24], *B. napus* (two uingenes) [43], *Millettia pinnata* (two unigenes) [24], *Pongamia pinnata* [20], and *Camelina sativa* [22]. However, FATB was not detected in the transcriptome of developing seeds in *Perilla frutescens* [27]. Both FATA and FATB (one and three unigenes, respectively) were detected in the *Camellia oleifera* seed transcriptome [25]. The absence of FATA in our study suggests a high production of saturated FAs in jojoba plastid with the major desaturation of FAs occurring in the ER.

LACS transfers the acyl group to CoA and transports the produced acyl-CoA into the ER for chain elongation and the generation of VLCFAs. LACS were highly expressed in developing jojoba seeds, indicated by the detection of 11 unigenes with an FPKM range from 3.21 to 95.33 (Table 2). In the *B. napus* transcriptome, ten unigenes were detected, three of which were upregulated and six were downregulated [43]. A family of six LACS genes was detected in the *Millettia pinnata* transcriptome with different levels of expression [24]. Also, LACS kept a high level of expression during the seed development of *Pongamia pinnata* [20], *Perilla frutescence* [27], and *Camelina sativa* [22].

### 3.3. Fatty Acid Desaturation

Various FA desaturases were detected in the developing jojoba seeds in both the plastid and ER. The ER-located enzymes included FAD2, FAD6, SLD, STE, and DES1 (SLD) with FPKM values of 69.07, 19.88, 33.31, 50.69, and 8.87, respectively (Table 2). The plastid-located desaturases included FAD4, FAD5, FAD6, and SAD6, with FPKM values of 2.05, 33.65, 8.42, and 1369.99, respectively. SAD6 converts stearoyl-ACP to oleoyl-ACP by the introduction of a *cis* double bond between carbons 9 and 10 of the acyl chain. Most of the plastid desaturases had a fairly low expression, except for SAD6, which had one of the highest expression levels in this study, indicating that it is the predominant active desaturase in developing jojoba seeds. ER-located enzymes had a fairly high expression, suggesting high desaturation activity in developing jojoba seeds. In the developing seeds of *B. napus*, FAD2, FAD3, FAD5, FAD6, FAD7, and FAD8 were detected at an upregulated level, whereas no SADs were detected [43]. FAD2 and FAD3 represented high expression level in the transcriptome of the developing seeds of the tree peony species *P. lutea* and *P. rockii* [26]. One unigene for the chloroplast FAD6 and FAD7/8 was detected with high expression in the transcriptome of the developing seeds of *Perilla frutescens* [27].

### 3.4. Fatty Acid Elongation

VLCFAs are synthesized by the repeated addition of 2C by a fatty acid elongase (FAE) complex. Three subunits of elongase KCS (FPKM, 865.4), KCR (FPKM, 402.24), and ECR (FPKM, 76.33) were highly expressed in developing jojoba seeds. On the other hand, the subunit HCD had low expression levels with an FPKM of 0.27 (Table 2). A high expression of elongase subunits indicates a high FA elongation activity, which is essential for the biosynthesis of VLCFAs and LCFAs (the two precursors for wax ester biosynthesis). In *Arabidopsis*, the KCS9 homologue was involved in the biosynthesis of C24 from the C22 fatty acid. The KCS9 gene’s knockout mutations showed an accumulation of C20 and C22. Additionally, seed-specific FAE was involved in the synthesis of erucic acid in *B. napus* [44]. In rice, WSL4, a homologue of *Arabidopsis thaliana* (KCS6/CER6) was involved in the biosynthesis of VLCFAs, especially the conversion of C20 to C24 FAs. This was confirmed by the expression of WSL4 and the production of C24 FA in yeast. Wax biosynthesis was highly reduced in WSL4 mutants [45].

### 3.5. LCFA Biosynthesis

LCFA biosynthesis is the second most important step for wax ester biosynthesis, with VLCFA biosynthesis taking first place. LCFAs are produced in plants through acyl reduction and the decarboxylation pathways with an intermediate step of aldehyde production [46]. LCFA biosynthesis is carried out by the FAR gene, which converts VLCFA to LCFA without releasing aldehyde, as documented previously in the acyl reduction pathway [46]. Ten unigenes were detected in the jojoba transcriptome for FAR with FPKM values ranging from 0.56 to 1613.05. This enzyme was originally isolated from developing jojoba embryos and was able to produce fatty alcohol in *E. coli*. It also produced fatty alcohol in *B. napus,* directing some of its seed triglycerides to wax ester biosynthesis [33]. The WSD1 enzyme was also detected in our transcriptome. It has wax ester synthase/diacylglycerol acyltransferase dual function. This was confirmed using wsd1 mutants that had reduced stem wax. The in vitro recombinant of the enzyme showed that WSD1 had ad 10 times wax synthase as its diacylglycerol acyltransferase activity and revealed the accumulation of wax ester in *Saccharomyces cerevisiae* [5]. LCFAs are converted to VLCFAs by oxidation through the activity of FAO, which was expressed in the jojoba transcriptome with an FPKM value of 39.02. FAR and FAO maintain an optimum concentration of VLCFAs and LCFAs for wax ester biosynthesis in developing jojoba seeds.

### 3.6. Triglyceride (TGA) Metabolism

The main five enzymes for TGA biosynthesis, G3PDH, GPAT, LPAAT, PAP, and DGAT, were represented in the jojoba transcriptome with FPKM values of 60.72, 13.59, 125.06, 24.62, and 16.53, respectively (Table 2). In *B. napus* seeds, various numbers of unigenes were detected for this group of enzymes. For example, 12, 11, 6, 2, and 1 unigenes were detected for G3PDH, GPAT, LPAAT, PAP, and DGAT, respectively [43]. A group of 7 unigenes were detected for GPAT, 8 unigenes for LPAAT, 4 unigenes for PAP, and 10 unigenes for DGAT in the *Millettia pinnata* transcriptome [24]. A high expression of TAG biosynthesis enzymes (GPAT, LPAT, PAP, DGAT, and PDAT) was positively correlated with oil accumulation during the seed development of *Pongamia pinnata* [20]. GPDH, LPAAT, and PAP maintained a high expression during seed development of the peony tree species *P. lutea* and *P. rockii*, whereas DGAT was higher in the developing seeds of *P. lutea* [26]. Unigene for GPAT, LPAT, PAP, and DGAT were detected in the *Perilla frutescens* seed transcriptome at expression levels consistent with the expression levels of FA biosynthesis genes [27]. In the *Camellia oleifera* seed transcriptome, six unigenes for PDAT (three PAP, three DGAT, one GPAT, and one LPAAT) were detected at a high level of expression during seed development [25]. They were also detected in the transcriptome of the developing seed of *Camelina sativa* [22].

TAGs are degraded by three lipases, including MAGL, DAGL, and TAGL (SDP). They were represented in the jojoba transcriptome with FPKM values of 15.34, 10.15, and 32.09, respectively (Table 2). Twelve unigenes were detected for TAGL and three were detected for MAGL in the *B. napus* transcriptome; these are essential for lipid degradation [43]. SDP1 (TAGL) was detected in the transcriptome of *Camelina sativa* developing seeds [22]. SDP regulates the TAG quantity and the TAG fatty acid composition of seed oil during seed development. Manipulation of SDP provides a potential approach for enhancing the quantity and quality of jojoba seed oil.

### 3.7. Phospholipid Metabolism

In our transcriptome, CDIPT, PSS, PES, PDCT, and CTP (CDS) were highly represented, with FPKM values of 24.10, 7.99, 6.6, 35.98, 11.93, and 20.11, respectively (Table 2). CDIPT catalyses the formation of PI from CDP-DAG. In a previous study, CDIPT was cloned from *Arabidopsis thaliana* and expressed in *E. coli* to produce PI from CDP-DAG and myo-inositol substrates; this reaction is reversible, indicating CDIPT must act to create balance and maintain the optimal concentration of myo-inositol in the cell [47]. Also, PSS was isolated from *Arabidopsis* and produced in *E. coli* confirming that it is essential for PS biosynthesis [48]. PES from *Arabidopsis* has been isolated and characterised. It was expressed in *E. coli* and proved that *E. coli* accumulated PE, but to our knowledge, it has not been detected in any other oil seed transcriptome [49]. PDCT unigenes were detected in the *B. napus* transcriptome [43]. PDCT represented high expression in *P. lutea* and *P. rockii*, but the level was higher in *P. lutea* [26]. PDCT expression showed a bell-shaped pattern during seed development in the *Perilla frutescens* seed transcriptome [27]. It was also detected in the transcriptome of the developing seed of *Camelina sativa* [22]. PDCT maintains the balance of free fatty acids and TAG by regulating the interconversion between PC and diacylglycerol (DAG) during the biosynthesis of TAGs. CTP (CDS) is encoded by five genes in *Arabidopsis*. CDS1, CDS2, and CDS3 are located in the ER membrane and have a similar function. Double mutants of CDS1 and CDS2 showed an accumulation of PA and a drastic decrease in PI level, leading to defects in cell division and expansion [50].

Phospholipases also maintain the balance between free fatty acids and TAGs. We detected six types of phospholipases—PLA1, PLA2, PLC, PLD, PLSGR2, and BDG3—with FPKM values of 3.8, 159.08, 35.9, 127.73, 13.24, and 42.11, respectively (Table 2). PLC and PLD showed a similar high expression in both tree peony species, *P. lutea* and *P. rockii,* during seed development and oil accumulation. In the same study, PLA2 was highly expressed during seed development in *P. rockii*, but, interestingly, its transcripts were not detected in the developing seeds of *P. lutea* [26]. PLA2 was detected in castor endosperm and may contribute to acyl editing [41]. In the *Camellia oleifera* seed transcriptome, 11 unigenes for PLD and eight for PLC were detected with high expression during seed development [25]. PLSGR2 is a phospholipase-like protein detected in *Arabidopsis* that is involved in the early development of gravitropic response and functions in a way similar to PLA-type proteins [51]. Putative BDG3 lysophospholipase has been detected in *Zea mays* (maize) [52].

### 3.8. Wax Ester Biosynthesis

Two wax ester synthases were detected in our transcriptome: WS and WSD1 with FPKM values of 101.99 and 32.29, respectively (Table 2). Although WS was first isolated from jojoba, it was not functionally expressed in other organisms, including *E. coli* and *S. cerevisiae* [5,53]. WSD1 is a bifunctional wax ester in *Arabidopsis thaliana*. It works as a WS/diacylglycerol acyltransferase (DAGT) in the ER membrane [5]. WSD1 was detected in the transcriptome of *Camelina sativa* developing seeds [22]. The presence of both WS and WSD1 in the transcriptome of the developing jojoba seeds indicates that during seed development, a high metabolic rate is directed toward biosynthesis of wax ester or DAG. WSD1 could also play a major role in maintaining the balance between wax ester and TAGs in jojoba seeds.

### 3.9. Lipid Transfer and Storage Proteins/Enzymes

The five detected lipid transfer and storage proteins included ACP, ACBP, nsLTPs, DIR1 (LTP), and OL. They were highly represented in the jojoba transcriptome with FPKM values of 1036.72, 244.44, 9748.18, 5214.36, and 3779.71, respectively (Table 2). Interestingly, nsLTPs (FPKM 9748.18), LTP (FPKM 5214.36), and OL (FPKM 3779.71) had the highest expression levels in this study. ACP binds and carries the growing acyl chain during fatty acid biosynthesis. ACBP is a 10 kDa protein that strongly binds medium and long chain acyl-CoA esters and contributes to their intracellular transport. Fifteen unigenes encoding ACBP were detected in the transcriptome of the developing seeds of *B. napus*, of which eight were downregulated in seeds compared to leaves [42,43]. nsLTPs are uniquely detected in land plants. They are small proteins (6.5–10 kDa) that have the ability to bind various hydrophobic molecules including fatty acids, phospholipids, and fatty acyl-CoA. They play an important role in the intracellular movement of membrane lipids. In addition, they contribute to various biological processes in plants, including cuticle and suberin biosynthesis, plant growth and development, fertilization, seed development and germination, and the response to biotic and abiotic stresses [54,55,56,57]. They also work as antimicrobials against pathogens (fungal, bacterial, or viral) by perturbing the integrity and function of the pathogen’s biological membranes [58,59]. LTPs are highly conserved, small (7–9 kDa) proteins detected only in higher plants [60,61]. They greatly contribute to the transfer of lipids, including phospholipids and fatty acids, through membranes [62]. They are divided into two subfamilies based on their size: LTP1 (9 kDa) and LTP2 (7 kDa) [63]. Different LTPs bind different lipid molecules, including fatty acids (C10–C18), acyl-CoAs, phospholipids, sterols, and various drugs [61]. Oleosins are small proteins that bind triglycerides and wax esters and facilitate their storage in the oil bodies. They have a hydrophilic oil body-binding domain between two amphipathic domains that contribute to oil body stabilization [43]. The *B. napus* transcriptome had 16 unigenes for oleosins. In addition, three homologues of the *Arabidopsis* oleosin (ole1, ole2, ole4) were detected in the transcriptome of *Camelina sativa* developing seeds. Ole1 had five-fold of the level of ole2 and ole4 expression [22].

## 4. Materials and Methods

Jojoba *(Simmondsia chinensis*, Link) seeds were collected at different stages of seed development from jojoba plants growing under normal Saudi Arabian conditions in Taif, Saudi Arabia. Seeds were collected at seven different developmental stages (1–7) based on their size (starting from 0.1 g as an early stage to 0.8 g as a late developmental stage). The seeds in each stage were then lyophilized at −58 °C for 48 h and then ground to a fine powder. Equal amounts of each stage of lyophilized seeds were pooled and used for RNA isolation.

### 4.1. Purification of mRNA

The total RNA was isolated from the developing seed powder mixture using RNeasy plant Mini Kit (Qiagen, Hilden, Germany) according to the manufacturer’s instructions. RNA samples with a RNA integrity number (RIN) of more than 7 were used for subsequent analysis. PolyA mRNA was purified using oligo-dT linked to magnetic beads for two cycles of purification. During the second elution step, polyA mRNA was fragmented and primed with a random hexamer for cDNA synthesis.

### 4.2. Synthesis of cDNA Libraries

cDNA libraries were constructed using TruSeq Stranded mRNA LT Sample Prep Kit following the manufacturer’s instructions (Illumina, California, United States). The fragmented, primed mRNA was reverse transcribed into first strand cDNA. Actinomycin D was included during first-strand synthesis to improve strand specificity by allowing RNA-dependent synthesis and to prevent DNA-dependent synthesis. The cDNA was purified using AMPure XP beads along with a magnetic stand. The blunt 3′ end of the cDNA was adenylated to prevent ligation to each other and enhance adapter ligation. Adapters (which have an extra T at the 3′ end) were ligated to both ends of the cDNA to enable them to hybridize on a flow cell. Ligated fragments were cleaned using AMPure XP beads. Indexed fragments were enriched by PCR amplification with adapter-specific primers and AMPure XP beads.

The quality of the cDNA libraries was checked using the DNA 1000 chip on the Agilent 2100 Bioanalyzer (Santa Clara, California, United States). The size and purity of the sample was expected to produce a band at about 260 bp. The libraries were quantified using qPCR according to the Illumina Sequencing Library qPCR Quantification Guide. The DNA libraries were normalized to 10 nM and pooled in equal volumes by the addition of 10 μL of each normalized library.

### 4.3. Sequencing and Sequence Analysis

The pooled libraries were sequenced on the NovaSeq 6000 System using NovaSeq 6000 S4 Reagent Kit (Illumina, California, United States) to generate 100 × 10^6^ paired end reads of 100 bp with a sequence depth of 12Gb per sample. The obtained raw FastQ sequences were checked for various quality parameters, including the overall read’s quality, the total bases, the total reads, GC%, and Q30 (% of bases with a quality Phred score over 30). Raw FastQ files were trimmed using the Trimmomatic program [64] to remove the adapter sequence, bases with a quality less than 3 from the read ends, the bases of reads that do not qualify for window size 4 and mean quality 15, reads with a length less than 36 bp, low-quality reads, contaminant DNA, and PCR duplicates. The trimmed reads of all samples were merged into one FastQ file to construct a transcriptome reference. Merged data were assembled using the Trinity program [65] and used for de novo transcriptome assembly. Transcripts above 36 bp were assembled into contigs (combining read sequences of a certain length of overlap to form longer fragments without N gaps). The longest contigs were clustered into non-redundant transcripts (unigenes) using the CD-HIT-EST program [66,67]. The unigenes obtained were used for the subsequent annotation, the open reading frames (ORF) prediction, and the expression analysis.

### 4.4. ORF Prediction and Abundance Estimation

ORFs with at least 100 amino acids were extracted and predicted for unigenes using TransDecoder (https://github.com/TransDecoder/TransDecoder) to identify the candidate coding regions within the transcript sequence. The trimmed reads for each sample were aligned to the assembled reference using the program Bowtie. For the differentially expressed gene analysis, the abundance of unigenes across the samples was estimated as a read count to indicate an expression figure using the RSEM algorithm [68]. The expression level was calculated as read count and as fragments per kilobase of transcript per million mapped reads (FPKM).

### 4.5. Functional Annotation

The unigenes were searched for in the Kyoto Encyclopedia of Genes and Genomes (KEGG), NCBI Nucleotide (NT), Pfam, Gene Ontology (GO), NCBI non-redundant Protein (NR), UniProt and Evolutionary genealogy of genes: Non-supervised Orthologous Groups (EggNOG) using BLASTN of NCBI BLAST [69] and BLASTX of the software DIAMOND [70] with an E-value default cut-off of 1.0 × 10^−5^.

## 5. Conclusions

The de novo transcriptome analysis revealed three specific features of Jojoba transcriptome during seed development: the detection of malonyl-CoA ligase (MCAL) with high FPKM value of 138.28, the detection of both WS and WSD1, and the detection of FATB with the absence of FATA. These enzymes substantiate general lipid biosynthesis, specifically wax ester biosynthesis. Also, high expression of FA biosynthesis (ACP, ACBP), lipid transfer (nsLTPs, LTPs, and lipid storage proteins in oil bodies (OL) was used to establish the wax ester synthesis pathway in developing Jojoba seeds. The results of this study will pave the way for innovative biotechnology approaches using these enzymes to improve jojoba wax ester production.

## Figures and Tables

**Table 1:** Summary of EggNOG annotation count and ratio for functional groups.

Category	Count	Ratio	Name
Information storage and processing			
J	797	2.19%	Translation, ribosomal structure and biogenesis
A	156	0.43%	RNA processing and modification
K	1462	4.03%	Transcription
L	7802	21.48%	Replication, recombination and repair
B	152	0.42%	Chromatin structure and dynamics
Cellular processes and signaling			
D	229	0.63%	Cell cycle control, cell division, chromosome partitioning
Y	1	0.0%	Nuclear structure
V	284	0.78%	Defense mechanisms
T	1542	4.25%	Signal transduction mechanisms
M	317	0.87%	Cell wall/membrane/envelope biogenesis
N	6	0.02%	Cell motility
Z	274	0.75%	Cytoskeleton
W	0	0%	Extracellular structures
U	866	2.38%	Intracellular trafficking, secretion, and vesicular transport
O	1612	4.44%	Posttranslational modification, protein turnover, chaperones
Metabolism			
C	504	1.39%	Energy production and conversion
G	1031	2.84%	Carbohydrate transport and metabolism
E	778	2.14%	Amino acid transport and metabolism
F	183	0.5%	Nucleotide transport and metabolism
H	211	0.58%	Coenzyme transport and metabolism
I	390	1.07%	Lipid transport and metabolism
P	407	1.12%	Inorganic ion transport and metabolism
Q	446	1.23%	Secondary metabolites biosynthesis, transport and catabolism
Poorly characterized			
R	0	0%	General function prediction only
S	16,866	46.44%	Function unknown

**Table 2:** Lipid biosynthesis enzymes detected in jojoba transcriptome during seed development.

Contig	Read Count	FPKM	Enzyme Name	Abbreviation	Location
**Fatty acid biosynthesis**					
c85820_g3_i2	7804	138.28	malonate-CoA ligase	MCAL	Cyt
c106491_g2_i1	18,389	88.75	acetyl-CoA carboxylase	ACCase- α	Pt
c106398_g2_i1	9264	195.99	biotin carboxylase 2	BC	Pt
c105364_g14_i1	6138	229.71	biotin carboxyl carrier protein of acetyl-CoA carboxylase	BCCP	Pt
c100625_g1_i2	178	4.95	acetyl-CoA carboxylase beta subunit	ACCase- β	Pt
c103501_g4_i1	6386	152.71	malonyl CoA-acyl carrier protein transacylase	MCAAT	Pt
c90813_g1_i1	13,507	264.77	3-oxoacyl-[acyl-carrier-protein] synthase I	KAS I	Pt
c88978_g1_i1	7348	100.10	3-oxoacyl-[acyl-carrier-protein] synthase II	KAS II	Pt
c93468_g1_i1	10,261	198.16	3-oxoacyl-[acyl-carrier-protein] reductase	KAR4	Pt
c89737_g1_i1	7589	254.27	3-hydroxyacyl-[acyl-carrier-protein] dehydratase	HAD	Pt
c92699_g1_i1	10,393	174.01	enoyl-[acyl-carrier-protein] reductase	EAR	Pt
c102110_g4_i1	492	21.47	palmitoyl-acyl carrier protein thioesterase	AAT(FATB)	Pt
c97538_g2_i1	7486	95.33	long chain acyl-CoA synthetase	**LACS**	Pt
**Fatty acid desaturation**					
c101312_g2_i1	3494	69.07	delta(12)-acyl-lipid-desaturase	FAD2	ER
c95630_g1_i2	755	19.88	omega-6 fatty acid desaturase	FAD6	ER
c101742_g2_i1	1478	33.31	delta(8)-fatty-acid desaturase 2	SLD	ER
c100587_g1_i2	566	8.87	sphingolipid delta(4)-desaturase DES1	SLD	ER
c93084_g1_i1	1527	50.69	delta(7)-sterol-C5(6)-desaturase	STE	ER
c88645_g1_i1	53	2.05	fatty acid desaturase 4	FAD4	Pt
c94122_g2_i1	2097	33.65	palmitoyl-monogalactosyldiacylglycerol delta-7 desaturase	FAD5	Pt
c98080_g1_i5	538	8.42	omega-6 fatty acid desaturase	FAD6	Pt
c91818_g1_i1	54958	1369.99	stearoyl-[acyl-carrier-protein] 9-desaturase	SAD6	Pt
**Fatty acid elongation**					
c87821_g1_i1	53726	865.40	beta-ketoacyl-CoA synthase	**KCS**	ER
c78850_g1_i1	14207	402.24	very-long-chain 3-oxoacyl-CoA reductase 1	KCR	ER
c140576_g1_i1	2	0.27	very-long-chain (3R)-3-hydroxyacyl-CoA dehydratase	HCD	ER
c90092_g1_i1	4639	76.33	very-long-chain enoyl-CoA reductase	ECR	ER
**Fatty alcohol biosynthesis and oxidation**					
c99851_g1_i2	69378	1613.05	alcohol-forming fatty acyl-CoA reductase	FAR	ER
c95694_g1_i1	2554	39.02	long-chain-alcohol oxidase FAO1	FAO	ER
**Triglyceride biosynthesis**					
c92718_g1_i1	4782	60.72	glycerol-3-phosphate dehydrogenase	G3PDH	ER
c93714_g1_i3	696	13.59	glycerol-3-phosphate acyltransferase	GPAT	ER
c96688_g1_i1	6533	125.06	1-acylglycerol-3-phosphate O-acyltransferase	LPAAT	ER
c103123_g1_i1	2943	24.62	phosphatidate phosphatase	PAP	ER
c102225_g2_i1	1510	16.53	diacylglycerol O-acyltransferase	DAGT	ER
c93304_g1_i3	489	15.34	monoacylglycerol lipase	MAGL	ER
c96753_g3_i1	1050	10.15	Mono-/di-acylglycerol lipase	DAGL	ER
c103208_g4_i1	2523	32.09	triacylglycerol lipase 2	SDP2, TAGL	ER
**Phospholipid metabolism**					
c70471_g1_i1	1021	24.10	CDP-diacylglycerol--inositol 3-phosphatidyltransferase 1	CDIPT	Golgi
c102453_g1_i4	1210	7.99	CDP-diacylglycerol--serine O-phosphatidyltransferase 1	PSS	ER
c104411_g1_i1	147	6.60	N-acylphosphatidylethanolamine synthase	PES	ER
c89493_g2_i1	325	11.93	phosphatidylcholine:diacylglycerol cholinephosphotransferase 1	PDCT	ER
c99283_g3_i1	1134	20.11	phosphatidate cytidylyltransferase 1	CTP (CDS)	ER
c95703_g2_i1	161	3.80	phospholipase A1	PLA1	ER
c89578_g1_i1	3262	159.08	phospholipase A2	PLA2	ER
c99134_g1_i1	2344	35.90	phospholipase C	PLC	PM
c91314_g1_i1	9735	127.73	phospholipase D	PLD	
c104602_g2_i3	1426	13.24	phospholipase SGR2	PLSGR2	
c94956_g1_i2	2784	42.11	lysophospholipase BODYGUARD 3	BDG3	
**Wax ester biosynthesis**					
c94816_g1_i1	3440	101.99	long-chain-alcohol O-fatty-acyltransferase	WS, Jojoba	ER
c104303_g2_i3	1158	32.29	O-acyltransferase WSD1	WS, WSD1	ER
**Lipid transfer and storage**					
c90684_g1_i1	173325	9748.18	non-specific lipid-transfer protein	nsLTPs	Exc
c24977_g1_i1	38455	5214.36	lipid-transfer protein DIR1	LTP	Exc
c92005_g1_i2	96918	3779.71	oleosin 18.2 kDa	OL	Cyt
c86220_g1_i1	30925	1036.72	acyl carrier protein	ACP	Pt
c55316_g1_i1	34307	2454.44	Acyl-CoA-binding protein	ACBP	Cyt

Cyt = cytosol, EXC = extracellular, Pt = plastid, PM = plasma membrane, ER = Endoplasmic reticulum, NU = nucleus.

## Supplementary Materials

The following are available online at https://www.mdpi.com/2223-7747/9/5/588/s1, Table S1: Statistics of initial merged assembled contigs, Table S2: Statistics of merged assembly of transcriptome, Table S3: Statistics of ORF predicted unigene of the merged assembly, Table S4: Distribution of annotated unigenes among various databases.

## Abbreviations

Net energy balance:NEB;net energy ratio: NER; greenhouse gas: GHG; wax ester synthase: WS; fatty alcohol reductase: FAR; biological process: BP; cellular component: CC; and molecular function: MF; eukaryotic orthologous groups: KOG; clusters of orthologous groups: COGs; non-supervised orthologous groups: NOGs; endoplasmic reticulum: ER; acetyl-CoA carboxylase: ACCase; malonate CoA ligase: MCAL; malonyl CoA-acyl carrier protein transacylase: MCAAT; 3-ketoacyl ACP synthase I: KASI; 3-oxoacyl-[acyl-carrier-protein] reductase: KAR; 3-hydroxyacyl-[acyl-carrier-protein] dehydratase: HAD; enoyl-[acyl-carrier-protein] reductase: EAR; palmitoyl-acyl carrier protein thioesterase: AAT, FATB; biotin carboxylase (BC), carboxyl transferase α: CT-α; carboxyl transferase β: CT-β; biotin carboxyl carrier protein: BCCP; glycerol-3-phosphate dehydrogenase: G3PDH; glycerol-3-phosphate acyltransferase: GPAT; 1-acylglycerol-3-phosphate O-acyltransferase: LPAAT; phosphatidate phosphatase: PAP;diacylglycerol O-acyltransferase: DGAT; monoacylglycerol lipase: MAGL; mon-/di-acylglycerol lipase: DAGL.
