# Supplementary material for: Transcriptome Analysis of Jojoba (Simmondsia chinensis) during Seed Development and Liquid Wax Ester Biosynthesis
Source: Plants (Basel). 2020 May 4;9(5):588. doi: 10.3390/plants9050588 (PMC7284725; doi:10.3390/plants9050588)
Supplement: Supplementary file 1 [file plants-09-00588-s001.zip › Supplementary Material T1.docx]

**Supplementary Material T1**. Statistics of initial merged assembled contigs

|  | # of genes | # of transcripts | GC% | N50 | Avg. contig  length (bp) | Total assembled  bases (bp) |
| --- | --- | --- | --- | --- | --- | --- |
| Initial assembled contigs | 176,106 | 233,291 | 40.66 | 1,158 | 710.12 | 165,664,392 |
| Longest contigs | 176,106 | 176,106 | 40.04 | 800 | 589.01 | 103,727,640 |
| Unigene contigs | 167,684 | 167,684 | 39.90 | 830 | 600.96 | 100,770,670 |
