# Supplementary material for: Transcriptome Analysis of Jojoba (Simmondsia chinensis) during Seed Development and Liquid Wax Ester Biosynthesis
Source: Plants (Basel). 2020 May 4;9(5):588. doi: 10.3390/plants9050588 (PMC7284725; doi:10.3390/plants9050588)
Supplement: Supplementary file 1 [file plants-09-00588-s001.zip › Supplementary Material T2.docx]

**Supplementary Material T2**. Statistics of merged assembly of transcriptome

| Assembly | Merge | |
| --- | --- | --- |
|  | All transcript contigs | Only longest isoform per gene |
| Total trinity 'genes' | 176,106 | 176,106 |
| Total trinity transcripts | 233,291 | 176,106 |
| Percent GC | 40.66 | 40.04 |
| N90 | 283 | 256 |
| N80 | 403 | 330 |
| N70 | 578 | 432 |
| N60 | 829 | 578 |
| N50 | 1,158 | 800 |
| N40 | 1,541 | 1,140 |
| N30 | 1,988 | 1,619 |
| N20 | 2,557 | 2,238 |
| N10 | 3,440 | 3,184 |
| Maximum contig length | 17,658 | 17,658 |
| Minimum contig length | 201 | 201 |
| Median contig length | 394.0 | 348.0 |
| Average contig length | 710.12 | 589.01 |
| Total assembled bases | 165,664,392 | 103,727,640 |
