# Supplementary material for: Transcriptome Analysis of Jojoba (Simmondsia chinensis) during Seed Development and Liquid Wax Ester Biosynthesis
Source: Plants (Basel). 2020 May 4;9(5):588. doi: 10.3390/plants9050588 (PMC7284725; doi:10.3390/plants9050588)
Supplement: Supplementary file 1 [file plants-09-00588-s001.zip › Supplementary Material T3.docx]

**Supplementary Material T3: :** Statistics of ORF predicted unigene of the merged assembly.

| **Unigene/ORF** | **NO** | **%** |
| --- | --- | --- |
| Total Unigene | 167,684 | -- |
| **ORF Predicted Unigene** | **20,162** | **12.02** |
| Single ORF Predicted Unigene | 18,082 | 89.68 |
| Multiple ORF Predicted Unigene | 2,080 | 10.32 |
| **Number of ORF** | **22,579** | **--** |
| Complete | 13,123 | 58.12 |
| Internal | 2,111 | 9.35 |
| 5prime_partial | 5,813 | 25.75 |
| 3prime_partial | 1,532 | 6.79 |
