# Supplementary material for: Transcriptome Analysis of Jojoba (Simmondsia chinensis) during Seed Development and Liquid Wax Ester Biosynthesis
Source: Plants (Basel). 2020 May 4;9(5):588. doi: 10.3390/plants9050588 (PMC7284725; doi:10.3390/plants9050588)
Supplement: Supplementary file 1 [file plants-09-00588-s001.zip › Supplementary Material T4.docx]

**Supplementary Material T4**: Distribution of annotated unigenes among various databases

| Assembly | Merge | % |
| --- | --- | --- |
| Total Unigene | 167,684 | ------- |
| KO_EUK | 39,712 | 23.68% |
| NT | 16,067 | 9.58% |
| EggNOG | 36,462 | 21.74% |
| Pfam | 27,158 | 16.2% |
| NR | 39,338 | 23.46% |
| UniProt | 24,457 | 14.59% |
| GO | 29,297 | 17.47% |
| Overall | 41,357 | 24.66% |
